# Changes in Salivary Immunoglobulin A, Stress, and Burnout in a Workplace Mindfulness Intervention: A Pilot Study

**DOI:** 10.3390/ijerph19106226

**Published:** 2022-05-20

**Authors:** Rosa Martínez-Borrás, Jaime Navarrete, Miguel Bellosta-Batalla, Cristina Martínez-Brotóns, David Martínez-Rubio

**Affiliations:** 1 Department of Psychology, Faculty of Health Sciences, Universidad Europea de Valencia, 46010 Valencia, Spain; rosa@psicoforma.es (R.M.-B.); miguel@elartedeescuchar.es (M.B.-B.); cristina@psicoforma.es (C.M.-B.); 2 Psicoforma, Integral Psychology Center, C/Maestro Clavé, 3, 2º, 3ª, 46001 Valencia, Spain; 3 Institut de Recerca Sant Joan de Déu, 08950 Esplugues de Llobregat, Spain; 4 Teaching, Research and Innovation Unit, Parc Sanitari Sant Joan de Déu, 08830 St. Boi de Llobregat, Spain; 5 El arte de Escuchar, Psychotherapy and Mindfulness. C/Madre Teresa Jornet, 10, 46009 Valencia, Spain; 6 Excellence Research Network PROMOSAM (PSI2014-56303-REDT), 28029 Madrid, Spain

**Keywords:** workplace intervention, mindfulness-based intervention, burnout, work-related stress, salivary Immunoglobulin A

## Abstract

The purpose of this pilot study was to examine the effectiveness of a 6-week workplace mindfulness- and self-compassion-based intervention (MSCBI) on perceived stress, burnout, immune functioning (assessed with the biomarker Immunoglobulin A), self-compassion, and experiential avoidance compared to a Workplace Stress Management Intervention. Both interventions were contextual, i.e., they were carried out in the workplace setting and during working hours. We followed a randomised controlled trial study design. The total sample was composed of 24 employees of an automotive company. One-way analyses of covariance between groups revealed significant differences in post-intervention levels of perceived stress, salivary Immunoglobulin A (sIgA), emotional exhaustion, self-compassion, and experiential avoidance, after adjusting for pre-test scores. The results of this study have several implications. Firstly, it confirms that MSCBIs might be more effective than regular psychoeducational interventions for work-related stress and burnout treatment. Secondly, sIgA can be used to assess immune function state changes when MSCBIs are carried out. Furthermore, these results indicate that it is feasible to carry out MSCBIs within companies and during working hours, and that these interventions can help effectively manage stress and burnout associated with the work environment.

## 1. Introduction

In recent years, workers’ vulnerability to stress has increased due to psychosocial risk factors such as bigger workloads, time pressure, and unstable working conditions [1]. For these reasons, chronic work-related stress has increased in prevalence too and, nowadays, it is a public health problem in many industrialised countries [2]. European Union sources concluded that approximately 30% of European workers may experience high stress levels in their jobs, indicating strong growth in this type of occupational risk [3]. Furthermore, stressed and chronically ill employees are expensive, both in terms of increased health care costs and decreased productivity [4].

Chronic exposure to employment-related stressors can lead to what has been dubbed ‘burnout syndrome’. Three canonical symptoms of burnout syndrome include exhaustion, cynicism, and occupational inefficacy [5]. ‘Exhaustion’ is defined as a subjective feeling of ceasing to be able to meet any or all emotional demands at work. ‘Cynicism’ is the imposition of affective distance towards one’s employment, colleagues, and/or consumers. Finally, ‘inefficacy’ is the perception of one’s own occupational incompetence. From this perspective, burnout syndrome may be seen as a progressively developed process resulting from the use of the relatively ineffective coping strategies with which professionals try to protect themselves from work-related stress [6]. In this process, exhaustion is a symptom that would contribute to the appearance of the cold and inhumane treatment given to customers [7]. 

Studies about workplace interventions to prevent or treat stress and burnout have grown exponentially during the last decade [8]. Among them, mindfulness-based interventions (MBIs) seem a promising approach to psychological impairment in this field [8,9]. Bishop et al. [10] described mindfulness as consciously bringing one’s attention to experiences that occur in the present moment, while maintaining an attitude of openness, acceptance, and curiosity. It is essentially a cognitive skill, and it has been found to be improved through training. In this regard, MBIs typically comprise a mixture of mindfulness practices, such as secular meditation, psychoeducation, and group interaction to teach participants to become more aware of thoughts, feelings, and bodily sensations, while approaching these internal states with a non-judgmental curiosity [11]. 

At the moment, research about MBIs at the workplace has shown promising evidence about the relationship between mindfulness-based training and improvements in perceived stress, work-related stress, and burnout [11,12,13,14]. However, this research field remains in an early phase of exploration and is in need of [9,15]: (a) rigorous experimental randomized controlled trials (RCT), (b) contrasting mindfulness interventions with competing approaches, and c) a measurement approach not only based on subjective and self-reported methods. For these purposes, feasibility and pilot studies are needed in order to plan full-size RCTs. 

Finally, MBIs have evolved to incorporate modules of compassion, a construct that has gained great interest in the Western part of the world in recent decades [16]. In Western psychology, compassion is a complex construct that involves cognitive, affective, and behavioural characteristics motivating one to alleviate the suffering of another being [17]. Concretely, self-compassion seems to be a protective factor for workers at risk of developing work-related stress, though more research is needed to establish to what extent this feature recently included in MBIs produces beneficial effects [18,19]. 

Overall, high methodological studies are needed to research the effects of MBIs on work-related stress, to the extent possible, including objective measures, which are infrequently assessed in studies about mindfulness training at work [20]. Therefore, the aim of this study was to evaluate the effectiveness of a contextual intervention, a Mindfulness- and Self-Compassion-Based Intervention (MSCBI), compared to a Workplace Stress Management Intervention (WSMI) carried out on a sample of employees from an automotive company. The main outcomes were perceived stress, burnout, immune functioning, self-compassion, and experiential avoidance. Two groups were compared, which were the MSCBI group (employees who received the MSCBI, i.e., the experimental group) and the WSMI group (employees who received the WSMI, i.e., the control group). The hypothesis stated that employees who were randomly allocated to the MSCBI group would report greater improvements in stress, burnout, immune functioning, self-compassion, and experiential avoidance compared to those randomly allocated to the WSMI group.

## 2. Materials and Methods

### 2.1. Participants

The final sample was composed of 24 employees (41.7% women) of the company Yanfeng Global Automotive Interiors (Almussafes, Valencia, Spain). The age range was from 25 to 54 years old (M = 40.54; SD = 9.38). Based on self-reports, 25% of the sample were single, 54.2% were married, and 20.8% were divorced. Regarding occupation, 62.5% of the sample were entry-level employees, 29.2% intermediate employees, and 8.4% managers. Table 1 shows the sociodemographic information of the original study sample. Chi-square and *t* tests revealed no significant group differences in demographic characteristics between MSCBI and WSMI groups at baseline.

The inclusion criteria were: age higher than 18 years old, fluency in Spanish, and availability to attend all sessions of the intervention. Exclusion criteria included already participating in other MBI or work stress management programme.

### 2.2. Procedure

All study procedures were carried out in accordance with the Declaration of Helsinki and approved by the Human Research Ethics Committee of the Ethics Commission for Experimental Research of the University of Valencia (H1499537283854). Once consent was obtained from the company managers, the staff members of the company were given the opportunity to enrol in the intervention programme through internal communication media. Participation in the programme and research was voluntary. We followed a randomised controlled study design. Those employees interested in participating were invited to an informational meeting (*n* = 45), where the intervention programme was described. Then, 40 employees accepted the invitation and came to the meeting. After being informed about the study, they were asked for their signed informed consent. After that, participating employees completed baseline assessments and then were randomly assigned to the MSCBI or WSMI groups via a randomization software (Random Allocation Software 2.0; [21]). 

**Figure 1:**
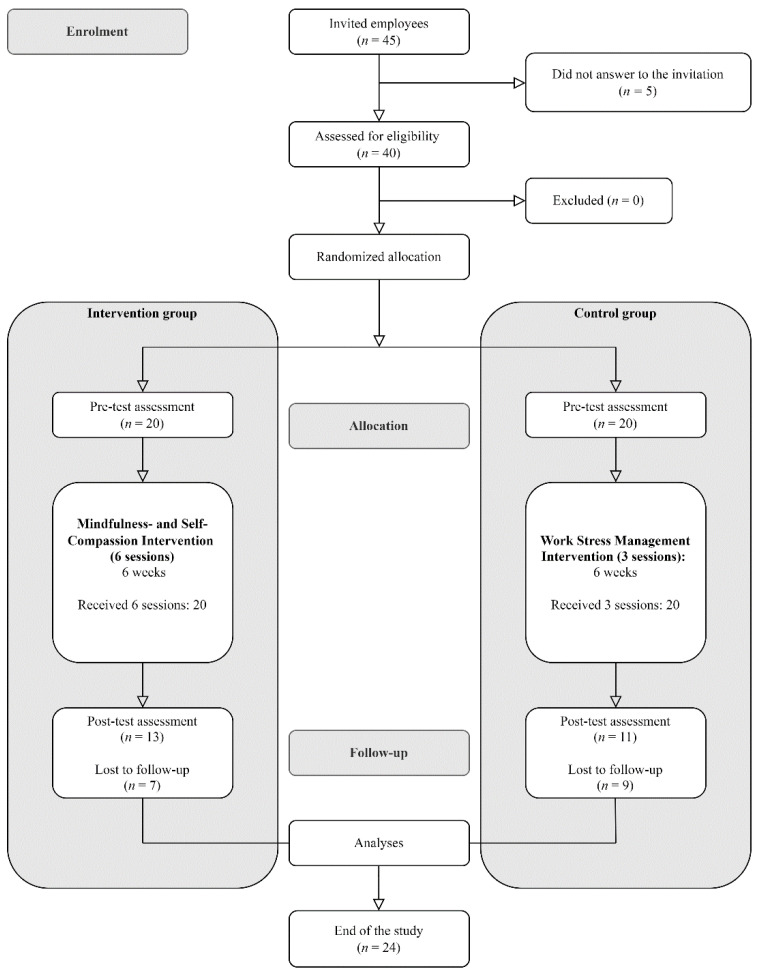
Flow of participants through the trial.

Those allocated to the MSCBI were divided into two groups to avoid having an excessively large intervention group. Both interventions were contextual, i.e., the MSCBI and WSMI were carried out in the workplace setting and during working hours. The assessment stages consisted of the baseline, pre/post first and last sessions (saliva samples), and post-programme assessments. Saliva samples were taken in the first and last sessions. For this purpose, participants were instructed to abstain from eating or drinking (except for water), as well as consuming caffeine, alcohol or any other drugs for 60 min prior to these sessions. Post-programme assessments were carried out a week after the last session. The participant flow chart is shown in Figure 1 below.

### 2.3. Mindfulness and Self-Compassion Programme

The MSCBI was a 6-week, 6-session programme led by an experienced mindfulness teacher and clinical psychologist who had previously conducted similar MBIs in workplace settings. It was carried out over a total of 12 contact hours during work time. A list of the contents and meditation practices for each session of the intervention is presented in Table 2. The MSCBI uses a variety of meditation practices and psychoeducation to foster mindfulness and self-compassion as resources that participants can then use to cope with work-related stress. It was developed specifically for the workers of the company in which this intervention was carried out.

The MSCBI is primarily experiential in nature. Throughout the programme, the instructor explained the theoretical framework of mindfulness and self-compassion and carried out exercises (including mindful breathing, a body scan, etc.). The sessions began with guided meditation, which was used as an aid to help the instructor explain the concept of mindfulness. All meditation practices performed in the sessions were followed by a discussion. Theoretical explanations and analyses of the difficulties that participants had each week were also part of all sessions. In the final session, the mindfulness teacher reviewed all of the modules covered and taught participants how to practice these skills outside the group setting.

### 2.4. Workplace Stress Management Intervention

The active control group participated in a 6-week, 3-session programme led by the same instructor that taught the experimental group. The WSMI was primarily theoretical in nature. The teacher explained aspects of psychoeducation regarding work-related stress and coping strategies. Participants were allowed to voluntarily share their personal experiences and received some feedback from the instructor and the rest of the group. These participants were on a waiting list and finally received the MSCBI. 

### 2.5. Measures

#### 2.5.1. Sociodemographic Information

Participants provided information on their age, gender (female, male), marital status (single, married, divorced, widowed), and work status (entry-level employees, intermediate employees, managers).

#### 2.5.2. Main Outcomes

Perceived Stress Questionnaire (PSQ [22]). The PSQ is a 30-item self-report instrument to measure ‘recent’ (during the last month) stress. Items are scored on a 4-point Likert scale (from 1 = almost never to 4 = almost always). A total score (possible range: 30–120) was calculated by adding up all items; higher scores indicate a higher perceived stress level. This measurement was taken during the pre- and post-intervention assessments. The validated Spanish version was used [23]. It showed an adequate internal consistency in the present study (Cronbach’s α ranging from 0.83 to 0.89).

Maslach Burnout Inventory-General Survey (MBI-GS [5]). The MBI-GS measures the three basic burnout dimensions: emotional exhaustion (MBI-EE), depersonalisation (MBI-D), and personal achievement (MBI-PA). It consists of 15 items measured on a 7-point Likert scale (from 0 = never to 6 = always). Higher scores indicate a higher level of burnout. This measurement was taken during the pre- and post-intervention assessments. The validated Spanish version was used [24]. It showed an adequate internal consistency in the present study (Cronbach’s α ranging from 0.78 to 0.87).

Salivary Immunoglobulin A (sIgA). Immunoglobulin A is an indicator of immune function state, as well as a reliable and widely used biomarker that interacts with stress response [25]. Ongoing and untreated work stress decreases levels of sIgA [26]. The levels of IgA were measured before and after the first and last sessions by collecting saliva samples from participants using Salivettes (Sarstedt, Rommersdolf, Germany). Saliva samples were frozen at 20 °C immediately after arriving at the laboratory and they were kept in this state until they were sent to an external laboratory for analysis. sIgA levels were measured by nephelometry (BN-II), using the reagent OSAR15 anti-IgA (Dade Behring), with a sensitivity of 0.2 mg/dL. 

Self-Compassion Scale, Short-Form (SCS-SF [27]). The SCS-SF is a 12-item self-report tool that assesses the three facets of self-compassion: Self-Kindness (SCS-SK), Common Humanity (SCS-CH), and Mindfulness (SCS-M). Items are scored on a 5-point Likert scale (from 1 = almost never to 5 = almost always). Following recent recommendations [28], a total score was calculated by adding the scores of items from the positive facets. Higher scores indicate a higher level of self-compassion (possible range: 6–30). The validated Spanish version was used [29]. It showed an adequate internal consistency in the present study (Cronbach’s α ranging from 0.63 to 0.84). This measurement was taken during the pre- and post-intervention assessments.

Acceptance and Action Questionnaire (AAQ-II [30]). The AAQ-II is a 7-item measure of psychological flexibility and experiential avoidance. Items are scored on a 7-point Likert scale (from 1 = never to 7 = always). Higher total scores mean both less flexibility and more experiential avoidance, and lower total scores mean greater flexibility and less experiential avoidance (possible range: 7–49). The validated Spanish version was used [31]. It showed an adequate internal consistency in the present study (Cronbach’s α ranging from 0.91 to 0.93). This measurement was taken during the pre- and post-intervention assessments.

### 2.6. Data Analysis

All analyses were performed using version 24 of IBM SPSS. First, the chi-square test and *t* tests were computed for categorical and continuous sociodemographic variables, respectively, to investigate statistical differences between groups at baseline. The scales’ internal consistency was established by calculating Cronbach’s alpha coefficients. Then, the normality of the distribution of scores for the dependent variables was determined by checking the kurtosis and skewness of the variables, performing the Kolmogorov–Smirnov Test, and inspecting the Normal Q-Q Plots. Moreover, Levene’s test for equality of variances was performed as part of the analyses. It was observed that all variables meet the assumption of normality, so paired-samples t-tests were conducted to evaluate the impact of the interventions on employees’ scores for the stress (PSQ), burnout (MBI-GS), self-compassion (SCS-SF), and experiential avoidance (AAQ-II). Then, a one-way analysis of covariance (ANCOVA) was conducted between groups to compare the effectiveness of both interventions. The independent variable was the type of intervention (MSCBI, WSMI), and the dependent variables consisted of PSQ, MBI-GS, SCS-SF, and AAQ-II scores assessed after the intervention was completed. Participants’ scores in the pre-intervention assessment were used as the covariates in this analysis. 

Regarding the biological variable, paired-samples t-tests were conducted to evaluate the impact of the first and last sessions of each intervention on employees’ immune function state (sIgA). Then, ANCOVAs were carried out to compare the effectiveness of the interventions through these sessions. For this purpose, we considered the type of intervention (MSCBI, WSMI) as the independent variable, post-session levels of sIgA as the dependent variable, and pre-session levels of sIgA as the covariates, for each session respectively.

## 3. Results

Table 3 displays the means and standard deviations of perceived stress, burnout, self-compassion, and experiential avoidance scores for each group. Based on the results, it is clear that the paired-samples t-tests conducted to evaluate the impact of the MSCBI showed a significant decrease in the scores for perceived stress, emotional exhaustion, depersonalisation, and experiential avoidance. The eta-squared statistic indicated large effect sizes ranging from 0.25 to 0.65 (except for the personal achievement subscale). Regarding the WSMI group, there were no statistically significant differences in perceived stress, burnout, and experiential avoidance scores. Furthermore, there was a statistically significant decrease in self-compassion scores from baseline to post-intervention assessments (see Table 3 for paired-samples t-tests). 

After adjusting for baseline scores, there were significant differences between the two intervention groups regarding post-intervention levels of perceived stress, emotional exhaustion, self-compassion, and experiential avoidance (see Table 3 for ANCOVAs). The partial eta-squared statistic indicated large effect sizes ranging from 0.24 to 0.42.

Table 4 shows sIgA means and standard deviations before and after the first and last session of each intervention and the results of the paired-samples t-tests. There were statistically significant increases in the sIgA levels of participants who underwent the MSCBI from the beginning to the end of the first and last sessions. The eta-squared statistic (0.66–0.67) indicated large effect sizes. Regarding the control group, only participants’ sIgA levels significantly increased in the last session, with a large effect size (0.64). Finally, after adjusting for pre-first session levels of sIgA, there were no significant differences between the two intervention groups regarding immunity function in the post-first session assessment (F_1, 22_ = 2.76, *p* = 0.112, *η_p_*^2^ = 0.121). However, there was a significant difference between groups in terms of post-last session sIgA levels (F_1, 22_ = 7.48, *p* = 0.013, *η_p_*^2^ = 0.272), after adjusting for pre-last session scores.

## 4. Discussion

The purpose of this study was to examine the effectiveness of a 6-week workplace mindfulness- and self-compassion-based intervention on perceived stress, burnout, immune functioning (assessed with the biomarker Immunoglobulin A), self-compassion, and experiential avoidance compared to an active group control.

Results on the differences between groups showed that participants in the MSCBI group saw a greater improvement in their average perceived stress and burnout (emotional exhaustion) than in the active control group (WSMI). These results are in line with recent studies on the effect of MSCBIs on work-related perceived stress and burnout, especially the exhaustion dimension, which is one of the most characteristic aspects of burnout [11,13]. Although there is not enough empirical evidence for any specific psychological interventions in the workplace [8], our results are in line with the evidence that workplace MBIs decrease burnout and stress levels and have other positive effects on mental health [32,33,34]. Further research should explain how these effects are produced, i.e., which are the involved mechanisms of action [9]. The present study measured psychological variables that might explain those effects, e.g., self-compassion and psychological flexibility. In this regard, mindfulness facets have been considered as mechanisms of change in the decrease of burnout after an MBI [35].

Moreover, participants who underwent the MSCBI saw a significant improvement in their average psychological flexibility and self-compassion. Previous studies have shown that psychological flexibility is highly correlated with self-compassion, and both are predictors of well-being (e.g., [36]). Furthermore, it has been described as an enhancer of self-compassion in work-related contexts [37]. In the same vein, although it is not possible to determine the effect of the improvement in self-compassion on the rest of the variables and other organisational outcomes, we think that it might explain the decrease in burnout and perceived stress due to the empirical evidence of the associations between those variables (e.g., [38]). In this regard, Reizer [39] found that self-compassion mediates the effect of attachment styles on job performance, organisational citizenship behaviours, turnover intentions, and emotional exhaustion. However, the role of compassion in organisational domains needs further consideration beyond hospital settings.

It seems that MBIs may also improve immune system functioning [40]. Bellosta-Batalla et al. [41] validated sIgA as a biological variable that is useful for evaluating the effectiveness of MBIs in field research. However, they did not take into account the influence of a control group in their study. Our results confirm that sIgA is a biomarker able to measure a change in immune function state among participants who have undergone an MBI. In this study, participants who underwent the MBI saw an increase in their immune function in the first and last sessions. This means that the MSCBI was able to counteract the effect of work-related stress on sIgA at least in the two sessions in which we could assess it. Apart from sIgA, C-reactive protein, interleukin, and antibody titers in response to the influenza vaccine are other biomarkers feasible for measuring immune function in workplace MBIs. Nevertheless, there is limited evidence of the effectiveness of mindfulness training in improving them [42].

Lastly, the limitations of this study should be taken into account. Firstly, some participants from both groups could not participate in the post-intervention assessment due to clashing schedules. As a result, the sample was smaller than expected which in turn might have underpowered the study. Secondly, although we followed a randomised clinical trial design, we were not able to properly register it. Finally, the assessment of sIgA in the first and last sessions was not enough to examine the effect of the interventions on it.

## 5. Conclusions

The results of this article have several implications. Firstly, the study increases the external validity of mindfulness- and self-compassion-based training in the work context and provides information to design an adequate and powered future RCT. Secondly, it suggests that MSCBIs might be more effective than regular psychoeducational interventions for work-related stress and burnout treatment. Thirdly, sIgA can be used to assess immune function state changes among participants who have undergone MBIs. However, more studies that assess this biomarker every session are needed to determine the effect of the intervention. Moreover, these results indicate that it is feasible to carry out MSCBIs within companies and during working hours and that these interventions might help to effectively manage stress and burnout associated with the work environment, which in turn has a beneficial influence on the biological parameters related to workers’ health.

## Figures and Tables

**Table 1:** Characteristics of study participants.

Baseline Characteristic	MSCBI Condition (*n* = 20)		WSMI Condition (*n* = 20)		Full Sample (*n* = 40)	
	**M**	**SD**	**M**	**SD**	**M**	**SD**
Age	41.18	9.43	42.83	11.23	41.93	10.17
	*n*	%	*n*	%	*n*	%
Gender						
Female	7	35	8	40	15	37.5
Male	13	65	12	60	25	62.5
Marital status						
Single	4	18.2	6	30	10	25
Married	10	50	10	50	20	50
Divorced	5	22.7	3	15	8	20
Widowed	1	4.5	1	5	2	5
Occupation						
Entry-level employees	10	50	10	50	20	50
Intermediate employees	8	40	7	35	15	37.5
Managers	2	10	3	15	5	12.5

**Table 2:** Sessions of Mindfulness- and Self-Compassion-Based program for employees.

Session	Program Topic	Meditations and Practices
Session 1	Motivation and basic concepts	Awareness of Breath Meditation Exploring the present experience
Session 2	Obstacles to practice Quiet the mind	Body Scan Acknowledgement journal
Session 3	Attention training	Mindfulness of thoughts Walking meditation
Session 4	The attitude of acceptance Relating to the experience	Acceptance and open-mindedness Listen carefully
Session 5	The relationship with oneself A kinder mind	Impartiality and kindness Self-care
Session 6	The relationship with others Compassion	Compassion (and self-compassion)

Note. Presented exercises are examples of those meditations and practices taught in each session.

**Table 3:** Means, standard deviations, paired samples t-test, and ANCOVA comparing primary and secondary outcomes.

Scale	MSCBI Condition		t (12)	*p*	*η^2^*	WSMI Condition		t (10)	*p*	*η^2^*	ANCOVA		
	Pre-Test	Post-Test				Pre-Test	Post-Test				F (1, 21)	*p*	*η_p_^2^*
PSQ	53.69 (11.30)	45.77 (12.13)	2.45	0.031	0.33	54.18 (13.13)	55.73 (12.38)	−0.79	0.448	0.06	6.64	0.018	0.24
MBI-EE	13.08 (7.38)	6.08 (5.63)	4.73	0.000	0.65	10.82 (4.98)	10.82 (5.19)	0.00	1.00	0.00	14.52	0.001	0.41
MBI-D	7.62 (3.99)	3.85 (3.05)	3.43	0.005	0.50	3.73 (2.61)	3.91 (2.74)	−0.26	0.800	0.01	1.61	0.219	0.07
MBI-PA	28.85 (4.76)	29.62 (5.77)	−0.62	0.547	0.03	26.36 (4.48)	27.64 (4.74)	−1.49	0.167	0.00	0.00	0.971	0.00
SCS-SF	9.56 (1.89)	10.42 (1.54)	−1.98	0.071	0.25	9.20 (1.65)	8.18 (1.96)	3.38	0.007	0.56	14.88	0.001	0.42
AAQII	21.00 (9.10)	17.23 (10.22)	2.52	0.027	0.35	18.55 (7.76)	22.00 (8.34)	−1.87	0.092	0.28	8.37	0.009	0.29

Note. Standard deviations are shown in brackets. PSQ = Perceived Stress Questionnaire; MBI-EE = Maslach Burnout Inventory-Emotional Exhaustion; MBI-D = Maslach Burnout Inventory-Depersonalisation; MBI-PA = Maslach Burnout Inventory-Personal achievement; SCS-SF = Self-Compassion Scale-Short Form; AAQII = Acceptance and Action Questionnaire.

**Table 4:** Results of paired-samples t-test for sIgA levels.

Immune Function (sIgA)	MSCBI Condition		t (12)	*p*	*η^2^*	WSMI Condition		t (10)	*p*	*η^2^*
	Pre-Test	Post-Test				Pre-Test	Post-Test			
First session	7.44 (3.89)	14.4 (8.26)	−4.81	0.001	0.66	7.07 (3.46)	9.89 (8.04)	−1.36	0.204	0.16
Last session	5.09 (2.69)	13.7 (7.14)	−4.95	0.000	0.67	5.38 (1.85)	8.30 (3.24)	−4.26	0.002	0.64

Note. Standard deviations are shown in brackets. sIgA = Salivary Immunoglobulin A.

## Data Availability

Data available on request from the authors.
